# A psychiatric mobile clinic in rural Ghana as a model to deliver professional services to a huge catchment area, review of 12 years experience

**DOI:** 10.1192/j.eurpsy.2023.749

**Published:** 2023-07-19

**Authors:** O. Shwarzman, B. Budde-Schwartzman

**Affiliations:** 1 Psychiatry, GYE NYAME MOBILE CLINICS, MAASE OFFINSO; 2 Gye Nyame Mobile Clinics, Maase- Offinso, Ghana

## Abstract

**Introduction:**

The population in remote areas in Ghana as in other low- and middle- income countries (LMIC’s) are known to suffer from limited access to quality mental health services. The challenges include limited inpatient and outpatient mental health services at the regional and district levels, shortage of well-trained professionals, poor funding by the government and difficulties for the patients to pay for medical costs, poor telecommunication services and the lack of adequate infrastructure.

**Objectives:**

We present a novel model of professional psychiatric mobile clinic, Gye Nyame Mobile Clinics, in remote areas in Ghana. This comprehensive service package connects the current loose ends of existing structural efforts in the subdistricts, trains regularly district hospital teams and bridges the gap between district hospital, primary health posts down to every patient.

**Methods:**

In this retrospective descriptive study we collected demographic data of all the patients who visited the Gye Nyame professional mobile clinic in Psychiatry (GNMC) from November 1, 2008 to October 31, 2019 in the ten health posts of Ghana’s Ashanti Region

**Results:**

Between November 2008 and October 2019, we counted 16.370 visits of patients with psychiatric/ neurological diagnosis. The patients suffered mostly from schizophrenia in 24,1%, general convulsions in 40,8 % and other psychotic disorders in 5,9% of the visits. 78,5% returned to our mobile clinic for follow-up, 100% could be treated on outreach.

**Conclusions:**

This community-based approach delivers psychiatric services to subdistrict and district levels and to patients who have no other access to these professional services. According to the results, a wide spectrum of pathologies and quantity of patients are seen – especially patients with no former treatment- the most common diagnosis in the rural area are schizophrenia, other psychotic disorders and generalized convulsions, followed by intellectual disabilities/autism spectrum disorder and cerebral malaria neuropsychiatric complications.

This is the first study to evaluate the implemented impact of integrated psychiatric services into existing structures in remote areas of LMIC`s.

**Disclosure of Interest:**

None Declared

